# The Relationship Between Peripheral Inflammatory Markers and High-Grade Cervical Lesions: A Retrospective Cohort Study

**DOI:** 10.3390/diagnostics15162107

**Published:** 2025-08-21

**Authors:** Aysun Alci, Necim Yalcin, Mustafa Gokkaya, Gulsum Ekin Sari, Harun Resit Turkmenoglu, Isin Ureyen, Tayfun Toptas

**Affiliations:** 1 Department of Gynecologic Oncology, Kahramanmaras Necip Fazıl City Hospital, Kahramanmaras 04600, Turkey; 2 Department of Gynecologic Oncology, Batman Training and Research Hospital, Batman 07200, Turkey; 3 Department of Gynecologic Oncology, Gaziantep City Hospital, Gaziantep 02700, Turkey; mugokkaya@gmail.com; 4 Department of Gynecologic Oncology, Health Scıences Unıversıty Antalya Training and Research Hospital, Antalya 07100, Turkey; drekingulsum@gmail.com (G.E.S.); harunturkmenoglu01@gmail.com (H.R.T.); isin.ureyen@gmail.com (I.U.); drttoptas@gmail.com (T.T.)

**Keywords:** cervical dysplasia, CIN2+, HPV 16, NLR, PLR, SII, SIRI

## Abstract

**Background/Objectives**: This study was designed to investigate the relationship between peripheral hematological inflammation markers, namely, neutrophil/lymphocyte ratio (NLR), platelet-to-lymphocyte ratio (PLR), monocyte/lymphocyte ratio (MLR), systemic immune inflammation index (SII), and systemic inflammatory response index (SIRI) and high-grade cervical lesions (CIN2+). **Methods**: A retrospective cohort analysis was conducted on 358 patients who underwent cervical excision procedures. Patients were divided into two groups: <CIN2 and CIN2+. Preoperative complete blood count data were used to calculate the inflammation indices. HPV genotypes were also recorded. Logistic regression and ROC analyses were performed to evaluate the predictive performance. **Results**: CIN2+ lesions were detected in 69.6% of participants. In the univariate analysis, only age and HPV 16 positivity (*p* < 0.005) showed a significant association with the presence of CIN2+. NLR, PLR, MLR, SII, and SIRI values did not show significant differences between groups (all *p* > 0.05). In the multivariate analysis, increasing age was independently associated with a decrease in the risk of CIN2+ (OR = 0.96, 95% CI: 0.94–0.99), while HPV 16 positivity was associated with an increase in risk (OR = 2.44, 95% CI: 1.43–4.18). ROC analysis showed that combining age and HPV 16 status improved the specificity (85.1%) of predicting CIN2+ compared to using age alone (42.2%). **Conclusions**: Peripheral haematological inflammation markers (NLR, PLR, MLR, SII, and SIRI) did not show predictive value in predicting CIN2+ lesions. However, age and HPV 16 infection were found to be independent predictors. These findings suggest that haematological indices may reflect systemic inflammatory responses but are not sufficient on their own for the detection of CIN2+. HPV genotyping is of critical importance for the early detection of high-grade lesions.

## 1. Introduction

Cervical cancer is the fourth most prevalent form of cancer among the female global population and constitutes the fourth most common cause of cancer-related fatalities. Consequently, it poses a grave threat to global public health [1]. The expansion of screening programmes and increased access to these programmes have significantly contributed to the early detection of cervical preinvasive lesions [2]. The most significant factor in the aetiology of the disease is persistent infection with high-risk human papillomavirus (HR-HPV) genotypes. However, additional risk factors such as immunosuppression and smoking also play a critical role in the development of cervical cancer [3]. The progression of the disease occurs through well-defined precancerous stages, known as cervical intraepithelial neoplasia (CIN), prior to the development of invasive carcinoma. These stages provide an important opportunity for early diagnosis and intervention [4]. Research has demonstrated that a considerable proportion of HPV infections and associated CIN lesions can be spontaneously resolved by the immune system, with clearance rates ranging from 60% to 90% [5].

In recent years, the concept of the tumour microenvironment (TME) has emerged as a prominent research topic in cancer biology. TME is a dynamic environment comprising tumour cells, surrounding stromal tissues, immune cells, and inflammatory components, and it plays a decisive role in tumour growth, spread, and response to treatment [6,7]. In this context, the importance of haematological markers for the evaluation of the inflammatory response has increased. A range of studies have explored the potential of various biomarkers, including the neutrophil/lymphocyte ratio (NLR), platelet-to-lymphocyte ratio (PLR), monocyte/lymphocyte ratio (MLR), systemic immune inflammation index (SII), and systemic inflammatory response index (SIRI), in facilitating the diagnosis and prognosis of a range of malignant and non-malignant diseases [8,9].

The objective of this retrospective cohort study is to evaluate the association between systemic inflammation markers, such as NLR, the PLR, MLR, SII, and SIRI, measured in peripheral blood samples from patients diagnosed with CIN and high-grade cervical preinvasive lesions. The objective of this study is to provide insights into the potential utility of these haematological parameters in early diagnostic processes.

## 2. Material and Methods

This study was conducted in accordance with the principles of the Declaration of Helsinki and was approved by the Ethics Committee of Antalya Training and Research Hospital (Approval No: 10/31, dated 19 June 2025). It involved a retrospective review of medical records from patients who presented to the Gynecologic Oncology Unit within the Department of Obstetrics and Gynecology at Antalya Training and Research Hospital between 2017 and 2025. Eligible participants were those who had undergone either a loop electrosurgical excision procedure (LEEP) or cold conization, and received a confirmed histopathological diagnosis. Patients whose excised cervical tissue showed only cervicitis or CIN 1 were categorized as Group 1. In contrast, those diagnosed histopathologically with CIN 2, CIN 3, high-grade squamous intraepithelial lesion (HSIL), adenocarcinoma in situ (AIS), or microinvasive squamous cell carcinoma were classified as Group 2 (CIN2+).

Peripheral venous blood samples were drawn from the antecubital vein using standard aseptic techniques and collected in ethylenediaminetetraacetic acid (EDTA)-containing tubes to prevent coagulation. Complete blood count (CBC) analyses were performed within two hours of collection using an automated hematology analyzer (e.g., Sysmex XN Series), following the manufacturer’s standard operating procedures and the institution’s internal quality control protocols.

Hematological inflammatory indices were calculated based on CBC parameters obtained prior to general anesthesia. These included NLR, typically ranging from 1.0 to 3.0; PLR, ranging from 0.78 to 3.53; and MLR, ranging from 100 to 300. SI was calculated as (neutrophils × platelets)/lymphocytes and expressed in 10^9^/L, with reference values between 0.19 and 0.42. Similarly, SIRI was calculated as (neutrophils × monocytes)/lymphocytes, with a reference range of 0.5 to 2.0 × 10^9^/L.

Patients without histopathological confirmation following LEEP, as well as those diagnosed with invasive cervical cancer who were referred for primary treatment, were excluded from the study.

## 3. Statistical Methods

The statistical analyses in this study were performed using SPSS (27.0, IBM Inc., Chicago, IL, USA). Normality was assessed using the Kolmogorov−Smirnov test, histogram analyses, skewness/kurtosis data, and Q−Q plot graphs. Quantitative variables were expressed as IQR (median [minimum–maximum]) or mean ± SD. Qualitative variables were expressed as frequency (N) and percentage (%). Relationships between two independent groups were examined using the Mann–Whitney U test or independent t-test. Relationships between qualitative parameters were examined using Fisher’s exact test. The effect profiles of variables on binary categories were evaluated using binary logistic regression analysis. The analysis of three-variable data was performed using the Spearman correlation test. Throughout the study, the type I error rate was set at 5% (α = 0.05), and a *p*-value < 0.05 was considered statistically significant at the 95% confidence level.

## 4. Results

In our study, the median age of participants was 43 years. The median values of inflammatory indices were as follows: NLR 1.94 (range: 0.69–7.34); PLR 117.39 (range: 48.07–886.36); MLR 0.23 (range: 0.09–0.90); SII 552.44 × 10^9^/L (range: 138.88–2783.18); and SIRI 1.05 × 10^9^/L (range: 0.31–5.29) (Table 1).

A total of 69.55% (*n* = 249) of participants were classified as CIN2+, while 30.45% (*n* = 109) were classified as <CIN2. In terms of menopausal status, 72.35% (*n* = 259) were premenopausal, 26.82% (*n* = 96) were postmenopausal, and 0.84% (*n* = 3) had unknown menopausal status. Cytological evaluation revealed that 26.82% (*n* = 96) had negative for intraepithelial lesion or malignancy (NILM), 8.38% (*n* = 30) had infection findings, and 13.13% (*n* = 47) had inadequate cervical cytology. Other cytological abnormalities included 9.50% (*n* = 34) ASCUS, 6.42% (*n* = 23) LSIL, 4.19% (*n* = 15) ASC-H, 7.54% (*n* = 27) HSIL, and 1.12% (*n* = 4) AGC. One patient (0.28%) was diagnosed with AIS, and no cases of endometrial cell degeneration or suspected invasion were reported. Notably, cytology results were unknown for 22.63% (*n* = 81) of patients. Regarding HPV status, 31.56% (*n* = 113) were HPV 16-positive, and 3.91% (*n* = 14) were HPV 18-positive. HPV 16+18 co-infection was detected in 3.63% (*n* = 13) of cases, while 15.08% (*n* = 54) were infected with HPV 16 along with other types, and 4.47% (*n* = 16) were infected with HPV 18 and other types. Only 0.84% (*n* = 3) had triple infections with HPV 16, 18, and other types. Additionally, 31.84% (*n* = 114) had infections with HPV types other than 16 and 18. A small proportion of patients (7.82%, *n* = 28) had unknown HPV status and 0.84% (*n* = 3) tested negative for HPV.

Statistically significant differences between the groups were observed exclusively in menopausal status and HPV genotype among all of the qualitative variables assessed (*p* = 0.006, *p* = 0.027) (Table 1).

The tables above summarize the findings of the logistic regression analyses exploring the relationship between the presence of CIN2+ lesions, hematologic inflammatory markers, and specific HPV genotypes. In the univariate analysis, age exhibited a statistically significant inverse association with CIN2+ status, indicating that older age may be linked to a reduced risk of developing high-grade cervical lesions (*p* = 0.006). In contrast, other inflammatory markers, namely, NLR, PLR, MLR, SII, and SIRI, along with HPV genotypes other than HPV 16, did not show significant predictive value in the univariate model (*p* = 0.0009) (Table 2). However, multivariate logistic regression identified both increasing age and the presence of HPV 16 infection as independent and statistically significant predictors of CIN2+ lesions (Table 3).

The receiver operating characteristic (ROC) analysis evaluated the predictive accuracy of age alone and the combined model of age plus HPV 16 positivity for the identification of CIN2+ lesions. The area under the curve (AUC) for age was 0.596, with a cut-off value of ≤47.5 years, yielding a sensitivity of 75.1% and a specificity of 42.2%, indicating modest discriminative ability. When HPV 16 status was added to the model, the AUC increased to 0.635, suggesting an improved predictive performance. Although the sensitivity decreased to 36.2%, the specificity significantly increased to 85.1%, demonstrating that the combined model is more effective at correctly identifying true negatives (Group 1). These results imply that HPV 16 positivity enhances the specificity of age-based predictions in detecting high-grade cervical lesions (Table 4).

**Figure 1:**
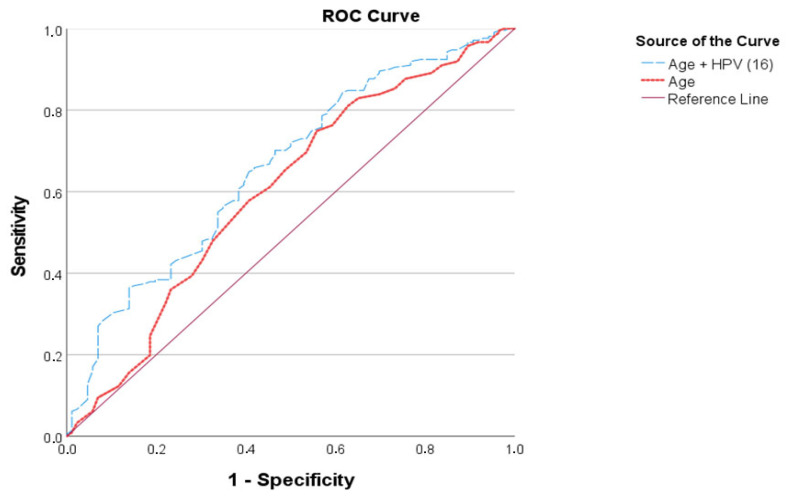
Comparative ROC curve: Age vs. age + HPV 16.

ROC curve demonstrating the diagnostic performance of age alone versus the combined model of age and HPV 16 positivity for predicting CIN2+ lesions. The red dotted line represents the predictive ability of age alone, while the blue dashed line represents the combined model. The diagonal solid line denotes the reference line indicating no discriminative ability (AUC = 0.5). The combined model (age + HPV 16) shows improved overall performance compared to age alone, as indicated by a higher AUC, suggesting better discriminative capacity in distinguishing CIN2+ cases from lower-grade lesions (Figure 1).

Spearman rank-order correlation was used to assess the monotonic relationship between the ordinal CIN group (CIN 1 < CIN 2 < CIN 3) and levels of inflammatory markers. None of the markers showed a statistically significant correlation with increasing CIN severity (*p* > 0.05) (Table 5).

## 5. Discussion

In this retrospective cohort study, we examined the relationship between CIN2+ lesions and various systemic hematological inflammatory indices, including NLR, PLR, MLR, SII, and SIRI. Our findings revealed that none of these markers were significantly associated with CIN2+ status. In contrast, multivariate logistic regression analysis demonstrated that increasing age had a protective effect (OR = 0.96, 95% CI: 0.94–0.99, *p* < 0.001), while HPV 16 positivity emerged as a significant independent risk factor (OR = 2.44, 95% CI: 1.43–4.18, *p* < 0.001). ROC analysis indicated that age alone had a modest discriminatory power (AUC = 0.596). However, when HPV 16 status was incorporated into the model, the AUC increased to 0.635, accompanied by a substantial rise in specificity (85.1%). These results suggest that although peripheral blood-derived inflammatory markers have limited utility for predicting CIN2+ lesions, combining HPV 16 status with age enhances predictive specificity for high-grade cervical lesions.

The prognostic value of systemic hematological markers remains inconsistent across the literature. A 2024 study involving 324 patients undergoing cervical biopsy reported that NLR, PLR, and SII levels were significantly higher in individuals without lesions compared to those with lesions. However, these markers demonstrated a poor discriminatory ability for high-grade lesions (AUC ≈ 0.57 for each), mirroring our own findings and underscoring their limited utility as standalone screening tools [10].

In another retrospective study involving 212 women, 106 patients had biopsy-confirmed CIN1–CIN3 lesions following LEEP, while the remainder served as healthy controls. Although no significant differences in NLR were observed between the control and CIN1 groups, there were statistically significant differences between CIN1 vs. CIN2 and CIN2 vs. CIN3. The authors proposed that NLR could serve as a staging-related marker reflecting CIN severity [11]. Unlike that study, we dichotomized cases into <CIN2 and CIN2+ groups, which may have limited our ability to detect stage-specific variations in inflammatory markers. Additionally, discrepancies in findings may reflect differences in sample size, patient demographics, and the heterogeneity of inflammatory responses.

A recent study by Wang et al. further contributed to this discussion. The authors compared NLR and PLR levels among three groups: cervical cancer (*n* = 42), HSIL (*n* = 31), and healthy controls (*n* = 31). While no significant differences were found between the cervical cancer and HSIL groups (NLR: *p* = 0.061; PLR: *p* = 0.759), both markers were significantly elevated in cervical cancer patients compared to healthy individuals (*p* < 0.001 for both). These findings imply that NLR and PLR may be more reflective of invasive disease rather than preinvasive stages [12]. In contrast, our study specifically excluded patients with invasive cervical cancer, which may explain the absence of significant differences in systemic markers. This suggests that elevated inflammatory indices could be a consequence of systemic immune activation in response to invasion.

Interestingly, a study by Bilir et al. found that NLR, PLR, MLR, and particularly SIRI were significantly elevated in patients with persistent HPV infection compared to those with transient infections. This indicates a potential role for SIRI in predicting viral persistence [13]. However, in our cohort, none of these markers were predictive of CIN2+ status, suggesting that their relevance may be limited to settings involving persistent infection, recurrence risk, or invasive disease rather than initial lesion detection.

Taken together, our results indicate that while systemic inflammatory markers may hold value in certain clinical contexts, they appear insufficient for accurately predicting high-grade preinvasive lesions when used in isolation. In contrast, HPV 16 positivity and patient age emerged as the most robust predictors of CIN2+ status.

The strength of our study lies in the inclusion of a large, single-center cohort, which enhances the statistical power and minimizes institutional variability. Furthermore, the binary categorization of <CIN2 and CIN2+ aligns with real-world clinical decision-making and reduces classification complexity. However, the study is not without limitations. Its retrospective design introduces inherent biases, and the lack of external validation limits the generalizability of the findings. This study has demonstrated that hematological inflammatory indices obtained from peripheral blood samples (including NLR, PLR, MLR, SII, and SIRI) have a limited predictive value for identifying CIN2+ lesions. This finding supports the idea that systemic inflammatory responses may become more pronounced in later, invasive stages of cervical neoplastic progression rather than in early, non-invasive stages such as CIN2. In conclusion, although these markers are widely used in clinical settings and are easily obtainable, their benefits appear limited in the context of preoperative counselling or clinical decision-making prior to LEEP procedures. Nevertheless, the findings from this study may provide a valuable foundation for future large-scale, prospective studies targeting diverse patient populations and more advanced stages of cervical pathology. Within such frameworks, the potential role of systemic inflammation in invasive disease progression could be further clarified. Additionally, the use of more sophisticated immunological profiling methods could enhance our understanding of the inflammatory mechanisms involved in cervical carcinogenesis. Specifically, the combined assessment of HPV 16 positivity and patient age could provide a more specific method for identifying individuals at risk of high-grade cervical lesions.

## Figures and Tables

**Table 1:** Distribution of qualitative and quantitative data.

		Frequency	Percentages (%)	Group 1 (n = 109, 30.4%)	Group 2 (n = 249, 69.6%)	*p* Value
Menopause	Unknown	3	0.84%	1 (0.92%)	2 (0.8%)	0.006 *
	Premenopausal ^†^	259	72.35%	67 (61.47%)	192 (77.11%)	
	Postmenopausal ^†^	96	26.82%	41 (37.61%)	55 (22.09%)	
Cytology	NILM	96	26.82%	31 (28.44%)	65 (26.1%)	0.276 *
	Infection	30	8.38%	6 (5.5%)	24 (9.64%)	
	Inadequate cervical cytology	47	13.13%	19 (17.43%)	28 (11.24%)	
	ASCUS	34	9.50%	7 (6.42%)	27 (10.84%)	
	LSIL	23	6.42%	5 (4.59%)	18 (7.23%)	
	ASC-H	15	4.19%	6 (5.5%)	9 (3.61%)	
	HSIL	27	7.54%	5 (4.59%)	22 (8.84%)	
	AGC	4	1.12%	2 (1.83%)	2 (0.8%)	
	Suspicion of invasion	0	0.0%	0 (0%)	0 (0%)	
	Endometrial degenerated cells	0	0.0%	0 (0%)	0 (0%)	
	Unknown	81	22.63%	28 (25.69%)	53 (21.29%)	
	AIS	1	0.28%	0 (0%)	1 (0.4%)	
HPV	Unknown	28	7.82%	9 (8.26%)	19 (7.63%)	0.027 *
	Negative	3	0.84%	1 (0.92%)	2 (0.8%)	
	HPV 16 ^†^	113	31.56%	20 (18.35%)	93 (37.35%)	
	HPV 18	14	3.91%	6 (5.5%)	8 (3.21%)	
	HPV 16 + 18	13	3.63%	5 (4.59%)	8 (3.21%)	
	HPV 16 and other types.	54	15.08%	21 (19.27%)	33 (13.25%)	
	HPV 18 and other types.	16	4.47%	5 (4.59%)	11 (4.42%)	
	HPV 16 + 18 and other types.	3	0.84%	2 (1.83%)	1 (0.4%)	
	Other HPV types.	114	31.84%	40 (36.7%)	74 (29.72%)	
	Minimum	Maximum		Distribution ^†^		
Age	27.0	68.0		43.0 (27.0 ± 68.0)		
NLR	0.69	7.34		1.94 (0.69–7.34)		
PLR	48.07	886.36		117.39 (48.07–886.36)		
MLR	0.09	0.9		0.23 (0.09–0.9)		
SII	138.88	2783.18		552.44 (138.88–2783.18)		
SIRI	0.31	5.29		1.05 (0.31–5.29)		

^†^ Subcategories with significant proportion differences between the groups are marked. * Fisher’s exact test. Data are presented as interquartile ranges (median, minimum–maximum) or mean ± SD. Abbreviations: AGC: atypical glandular cells; AIS: adenocarcinoma in situ; ASC-H: atypical squamous cells; ASCUS: atypical squamous cells of undetermined significance; HPV: human papillomavirus; HSIL: high-grade squamous intraepithelial lesion; LSIL: low-grade squamous intraepithelial lesion; NILM: negative for intraepithelial lesion or malignancy; NLR: neutrophil-to-lymphocyte ratio (ratio, unitless); PLR: platelet-to-lymphocyte ratio (ratio, unitless); MLR: monocyte-to-lymphocyte ratio (ratio, unitless); SII: systemic immune-inflammation index = (neutrophil × platelet)/lymphocyte (10^9^/L); SIRI: systemic inflammation response index = (neutrophil × monocyte)/lymphocyte (10^9^/L).

**Table 2:** Univariate logistic regression results.

Variables	B	Nagelkerke R2	*p*	OR	95% CI Lower Limit	95% CI Upper Limit
Age	−0.0367	−0.0083	0.0068	0.96	0.94	0.99
NLR	0.1883	−0.0019	0.2196	1.21	0.89	1.63
PLR	0.0021	−0.0011	0.3731	1.0	1.0	1.01
MLR	0.4717	−0.0002	0.7129	1.6	0.13	19.77
SII	0.0006	−0.0025	0.1593	1.0	1.0	1.0
SIRI	0.1437	−0.0007	0.4378	1.15	0.8	1.66
HPV 16	0.9001	−0.0132	0.0009	2.46	1.44	4.19
HPV 16 + others	−0.3625	−0.0015	0.2356	0.7	0.38	1.27
HPV 16 + 18	−0.2982	−0.0003	0.6082	0.74	0.24	2.32
HPV 16 + 18 + others	−1.4636	−0.0017	0.2341	0.23	0.02	2.58
HPV 18	−0.4898	−0.0009	0.3753	0.61	0.21	1.81
HPV 18 + others	−0.2581	−0.0003	0.6259	0.77	0.27	2.18
HPV others	−0.326	−0.002	0.1737	0.72	0.45	1.15
HPV_Negatif	−0.0643	−0.0	0.9583	0.94	0.08	10.45

Reference category in comparison: Group 1. LR: likelihood ratio; LL: log likelihood; CI: confidence interval; OR = odds ratios; ref = reference subcategory. Abbreviations: NLR: neutrophil-to-lymphocyte ratio (ratio, unitless); PLR: platelet-to-lymphocyte ratio (ratio, unitless); MLR: monocyte-to-lymphocyte ratio (ratio, unitless); SII: systemic immune-inflammation index = (neutrophil × platelet)/lymphocyte (10^9^/L); SIRI: systemic inflammation response index = (neutrophil × monocyte)/lymphocyte (10^9^/L).

**Table 3:** Multivariate logistic regression: HPV 16 and age.

Variables	B	Nagelkerke R2	*p*	OR	95%CI Lower Limit	95%CI Upper Limit
Age	−0.0364	−0.0207	*p* < 0001	0.96	0.94	0.99
HPV 16	0.893	−0.0207	*p* < 0001	2.44	1.43	4.18

Reference category in comparison: Group 1. LR: likelihood ratio; LL: log likelihood; CI: confidence interval; OR = odds ratio; ref = reference subcategory.

**Table 4:** ROC analysis summary for predictive models.

Model	AUC (95% CI)	Cut-Off	*p*	Sensitivity (%)	Specificity (%)
Age	0.596 (NA)	≤47.5	0.004	75.1	42.2
Age + HPV 16	0.635 (NA)	≥0.74	<0.001	36.2	85.1

AUC = area under curve; ROC = receiver operating characteristic; CI = confidence interval; Reference category = Group 1.

**Table 5:** Spearman rank correlation between the CIN group and inflammatory markers (CIN 1 → CIN 3 as ordinal).

Inflammatory Marker	Spearman ρ	*p*-Value
NLR	0.030	0.565
PLR	0.008	0.882
MLR	−0.029	0.586
SII	0.028	0.602
SIRI	−0.028	0.603

Spearman rank correlation abbreviations: NLR: neutrophil-to-lymphocyte ratio (ratio, unitless); PLR: platelet-to-lymphocyte ratio (ratio, unitless); MLR: Monocyte-to-Lymphocyte ratio (ratio, unitless); SII: systemic immune-inflammation index = (neutrophil × platelet)/lymphocyte (10^9^/L), SIRI: systemic inflammation response index = (neutrophil × monocyte)/lymphocyte (10^9^/L).

## Data Availability

The data generated in the present study may be requested from the corresponding author.

## Abbreviations

AGC	Atypical Glandular Cells
AIS	Adenocarcinoma In Situ
ASC-H	Atypical Squamous Cells—cannot exclude HSIL
ASCUS	Atypical Squamous Cells of Undetermined Significance
CBC	Complete Blood Count
CIN	Cervical Intraepithelial Neoplasia
HR-HPV	Human Papillomavirus
EDTA	Ethylenediaminetetraacetic Acid
HPV	Human Papillomavirus
HR-HPV	High risk Human Papillomavirus
HSIL	High-Grade Squamous Intraepithelial Lesion
LSIL	Low-Grade Squamous Intraepithelial Lesion
MLR	Monocyte-to-Lymphocyte Ratio
NILM	Negative for Intraepithelial Lesion or Malignancy
NLR	Neutrophil-to-Lymphocyte Ratio
PLR	Platelet-to-Lymphocyte Ratio
SII	Systemic Immune-Inflammation Index
SIRI	Systemic Inflammation Response Index
TME	Tumour Microenvironment
